# Theoretical Study of the Magnetic Mechanism of a Pca21 C_4_N_3_ Monolayer and the Regulation of Its Magnetism by Gas Adsorption

**DOI:** 10.3390/molecules29215194

**Published:** 2024-11-02

**Authors:** Dongqiu Zhao, Xiao Tang, Xueying Gao, Wanyan Xing, Shuli Liu, Huabing Yin, Lin Ju

**Affiliations:** 1 School of Physics and Electric Engineering, Anyang Normal University, Anyang 455000, China; dqzhao@aynu.edu.cn (D.Z.); 221101050@stu.aynu.edu.cn (X.G.); 221101039@stu.aynu.edu.cn (W.X.); 01958@aynu.edu.cn (S.L.); 2 College of Science, Nanjing Forestry University, Nanjing 210037, China; 3 Joint Center for Theoretical Physics, Institute for Computational Materials Science, School of Physics and Electronics, Henan University, Kaifeng 475004, China; yhb@henu.edu.cn

**Keywords:** magnetic mechanism, first-principles calculations, gas adsorption, two-dimensional materials

## Abstract

For metal-free low-dimensional ferromagnetic materials, a hopeful candidate for next-generation spintronic devices, investigating their magnetic mechanisms and exploring effective ways to regulate their magnetic properties are crucial for advancing their applications. Our work systematically investigated the origin of magnetism of a graphitic carbon nitride (Pca21 C_4_N_3_) monolayer based on the analysis on the partial electronic density of states. The magnetic moment of the Pca21 C_4_N_3_ originates from the spin-split of the 2*p*_z_ orbit from special carbon (C) atoms and 2*p* orbit from N atoms around the Fermi energy, which was caused by the lone pair electrons in nitrogen (N) atoms. Notably, the magnetic moment of the Pca21 C_4_N_3_ monolayer could be effectively adjusted by adsorbing nitric oxide (NO) or oxygen (O_2_) gas molecules. The single magnetic electron from the adsorbed NO pairs with the unpaired electron in the N atom from the substrate, forming a N_sub_-N_ad_ bond, which reduces the system’s magnetic moment from 4.00 μ_B_ to 2.99 μ_B_. Moreover, the NO adsorption decreases the both spin-down and spin-up bandgaps, causing an increase in photoelectrical response efficiency. As for the case of O_2_ physisorption, it greatly enhances the magnetic moment of the Pca21 C_4_N_3_ monolayer from 4.00 μ_B_ to 6.00 μ_B_ through ferromagnetic coupling. This method of gas adsorption for tuning magnetic moments is reversible, simple, and cost-effective. Our findings reveal the magnetic mechanism of Pca21 C_4_N_3_ and its tunable magnetic performance realized by chemisorbing or physisorbing magnetic gas molecules, providing crucial theoretical foundations for the development and utilization of low-dimensional magnetic materials.

## 1. Introduction

Two-dimensional (2D) ferromagnetic materials, capable of atomic-level charge and spin control, are regarded as excellent potential candidates for future memory and logic applications [1]. The 2017 discovery of atomically thin vdW magnets, chromium(III) iodide (CrI_3_) [2] and chromium germanium telluride (CrGeTe_3_) [3], unveiled new opportunities for investigating unique magnetic phenomena in reduced dimensions. Since then, numerous 2D magnets have been discovered, including ferromagnets [4,5,6,7,8], antiferromagnets [9,10], and magnetic topological insulators [11]. These 2D magnets, exhibiting remarkable optical, magnetic, magneto-electric, and magneto-optic properties, are highly valuable for investigating new quantum phenomena in low-dimensional magnetism and for advancing the next generation of spintronic devices. These discoveries have inspired numerous theoretical investigations aimed at elucidating the fundamental mechanisms, predicting novel materials, and designing spintronic devices leveraging 2D magnets [12,13,14].

In addition to traditional 2D ferromagnetic materials based on transition metals or rare earth elements [15,16], substantial efforts have been focused on the exploration of metal-free 2D ferromagnetic materials, which are more cost-effective and environmentally friendly. However, due to the absence of localized spins, most of the metal-free materials are inherently diamagnetic. Therefore, advanced modifications of the electronic structure of non-metallic materials are needed so that they contain unpaired electrons and magnetic coupling capable of exhibiting ferromagnetism. Two-dimensional graphene has garnered significant attention as a novel electronic material due to its unique electrical properties [17]. It is expected that graphene and similar carbon-based 2D materials could be advantageous in spintronic applications. However, theoretical and experimental studies have demonstrated that most carbon-based 2D materials are inherently nonmagnetic [18]. To address this limitation, various strategies including doping [19,20], hydrogenation [21], and defect engineering [22,23] have been proposed to induce ferromagnetism. However, the application of graphene is also limited by its zero bandgap [24]. Graphite carbon nitride (g-C_3_N_4_), possessing a large bandgap and structurally similar to graphene, has been thoroughly studied as a typical 2D metal-free material due to its distinct optical and electronic properties, along with its strong chemical and thermal stability [25]. Numerous efforts have primarily concentrated on introducing magnetism into nonmagnetic 2D C_3_N_4_ through methods such as doping [26,27,28,29] and defect engineering [30]. However, achieving long-range magnetic order in the plane using these methods is limited and remains a significant challenge, because the magnetism induced by such doping and modifications is typically localized and lacks long-range magnetic order. Exploring intrinsic metal-free ferromagnetic materials holds promise for overcoming these shortcomings. In a recent breakthrough, a novel form of stable graphitic carbon nitride material (Pca21 C_4_N_3_ monolayer) has been forecasted through a randomized approach grounded in group and graph theory (RG2) [31]. Our previous theoretical study [32] revealed that the Pca21 C_4_N_3_ monolayer not only exhibited intrinsic long-range ordered ferromagnetism, but also had a high Curie temperature of 246 K. Additionally, the results of ab initio molecular dynamics (AIMD) simulations demonstrate that the Pca21 C_4_N_3_ monolayer exhibits excellent thermal stability.

Precious studies have revealed that molecular adsorption presents an efficient method for adjusting the electronic structures and magnetic properties of several 2D materials such as graphene [33], phosphorene [34], MXenes [35], and transition metal dichalcogenides [36]. As a newly discovered two-dimensional non-metallic ferromagnet, Pca21 C_4_N_3_ holds promise of expanded applications by tuning its electronic structure and magnetic properties through molecular adsorption. To the best of our knowledge, research on regulating the electronic structure and magnetic properties of the Pca21 C_4_N_3_ monolayer through the adsorption of polar molecules and non-polar molecules has not been reported yet. Herein, first-principles calculations have been employed to comprehensively examine the electronic structure and magnetic properties of both the Pca21 C_4_N_3_ monolayer and its gas-molecule-adsorbed counterparts.

## 2. Results and Discussion

### 2.1. Electronic and Magnetic Properties of Pristine Pca21 C_4_N_3_ Monolayer

As is known to all, a material’s microscopic structure determines its physical and chemical properties. Therefore, to explore the origins of its magnetic and electronic structures, the morphology structure of the Pca21 C_4_N_3_ monolayer is first investigated in detail. The optimized lattice parameters of Pca21 C_4_N_3_ are *a* = 4.16 Å and *b* = 4.77 Å. The ground-state Pca21 C_4_N_3_ monolayer exhibits a highly corrugated structure with a thickness of 1.28 Å, where nitrogen (N) atoms occupy both the top-most and bottom-most positions. This pronounced corrugation is likely caused by the lone pair interactions of nitrogen atoms. The Pca21 C_4_N_3_ monolayer features alternating 12-membered macrocycles and 6-membered microcycles. Both types of cycles are constructed from two kinds of covalent bonds, namely carbon-carbon (C-C) bonds and carbon-nitrogen (C-N) bonds. The lengths of the C-C bonds vary from 1.45 Å to 1.46 Å, averaging at 1.46 Å. Meanwhile, C-N bond lengths range between 1.34 Å and 1.36 Å, with an average of 1.35 Å. As displayed in Figure S1, each carbon (C) atom bonds with three other atoms. Carbon atoms bonded solely with three other carbon atoms are labeled as C_C_, whereas those bonded with one carbon and two nitrogen atoms are designated as C_N_. These two types of C exhibit different physical and chemical properties due to differences in their surrounding chemical environments. Additionally, each nitrogen atom is bonded to two C_N_ atoms. Furthermore, the Pca21 space group belongs to the orthorhombic system and exhibits chiral symmetry. It is characterized by only translational and rotational symmetry operations, with no inversion or mirror symmetry operations of any kind.

**Figure 1:**
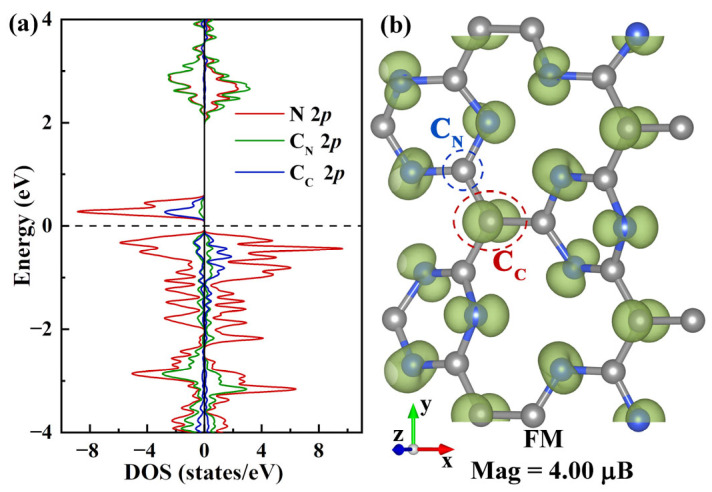
(**a**) The spin-polarized PDOS of C_C_, C_N_, and N 2*p* in the Pca21 C_4_N_3_ monolayer. The C_C_ 2*p*, C_N_ 2*p*, and N 2*p* states are represented by blue, green, and red lines, respectively. The Fermi level (*E*_f_) is indicated by a black dashed line and set to 0 eV. This representation of *E*_f_ is also applicable to the subsequent density of states (DOS) plots. (**b**) The three-dimensional isosurfaces (iso-value of 0.01 e/Å^3^) depicting net magnetization density (difference between spin-up and spin-down), which also applies to the subsequent net magnetization density, for the Pca21 C_4_N_3_ monolayer in the ferromagnetic state. Gray spheres symbolize C atoms, and blue spheres denote N atoms, which also applies to the subsequent Figures 2, 5 and 7. The subfigure labels represent the coordinates.

The Pca21 C_4_N_3_ monolayer is fundamentally a modified graphitic C_3_N_4_, where certain nitrogen atoms are substituted by carbon atoms. Given that one carbon atom has one fewer electrons than one nitrogen atom, the C_4_N_3_ system features unpaired electrons, which primarily contribute to the magnetism. The electronic structure properties near the Fermi level (*E*_f_) determine the fundamental physical and chemical properties of materials, such as photoelectric activity, catalytic activity, and magnetic performance. To investigate the origin of magnetic performance and the distribution of magnetic moments in the Pca21 C_4_N_3_ monolayer, we examined the electronic orbitals near the Fermi level of C_C_, C_N_, and N, as well as the spatial spin density distribution in the pristine monolayer. Figure 1a displays the spin-resolved partial density of states (PDOS) for Pca21 C_4_N_3_, indicating that the bandgap (*E*_g_) in the spin-down channel is 0.36 eV, whereas the one in the spin-up channel is much larger, reaching 2.24 eV. Near the Fermi level, the valence band (VB) for both the spin-up and spin-down channels is predominantly occupied by N 2*p* electrons, with a minor contribution from C_C_ 2*p* electrons, and the spin-up channel contains more electrons than the spin-down channel. The conduction band minimum (CBM) in the spin-down channel is mainly composed of N 2*p* states with a slight contribution from C_C_ 2*p* states, while the CBM in the spin-up channel consists equally of C_N_ 2*p* and N 2*p* states. Additionally, the PDOS displays a completely spin-polarized condition with a magnetic moment of 4.00 μ_B_. As a ferromagnetically stable Pca21 C_4_N_3_ monolayer [32], its magnetic performance mainly originates from the 2*p* states of C_C_ and nitrogen electrons, where all nitrogen electrons contribute more to the total magnetic moment than C_C_ electrons (Figure 1a). The distribution of PDOS near the band edge for the Pca21 C_4_N_3_ monolayer may be due to the atomic ratio of C_C_, C_N_, and N in its unit cell being 1:3:3, along with the presence of unpaired electrons and lone pair electrons on nitrogen atoms. The spatial spin density distribution provides detailed information on the magnetic moment distribution of the system in real space. As shown in Figure 1b, the spatial spin density of the Pca21 C_4_N_3_ monolayer reveals that the magnetic moments are mainly distributed on the 2*p* orbitals of nitrogen atoms and the 2*p*_z_ orbitals of C_C_ atoms, with almost no distribution on the C_N_ atoms. The magnetic moment distribution in this monolayer may, in turn, result from the different chemical environments around C_C_ and C_N_, as well as the influence of lone pair electrons on nitrogen.

Although the valence band electrons far from *E*_f_ and high-energy conduction band (CB) states have been less studied in depth, these electronic states, together with those near the band edge, collectively provide information on the orbital hybridization of constituent atoms and the bonding characteristics between atoms in the material. To investigate the origin of magnetic performance, it is essential to understand the electronic structure in the low-energy region of the VB and the high-energy region of the CB of Pca21 C_4_N_3_. Given the presence of unpaired electrons in pristine Pca21 C_4_N_3_, the spin-resolved PDOSs for the 2*s* and 2*p* states of N, C_C,_ and C_N_ in the Pca21 C_4_N_3_ monolayer have been considered.

**Figure 2:**
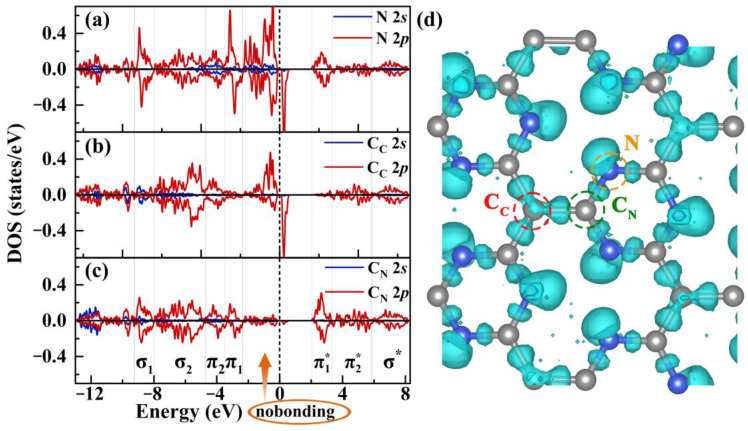
The spin-resolved PDOS diagrams for the 2*s* and 2*p* states of one (**a**) N, (**b**) C_C_, and (**c**) C_N_ atom in the Pca21 C_4_N_3_ monolayer. The 2*s* and 2*p* states are represented by blue and red lines, respectively. (**d**) The ELF of Pca21 C_4_N_3_ monolayer, with cyan regions indicating electron accumulation. The isosurface value is set to 0.60 e/Å^3^.

Based on the corrugated microscopic structure of Pca21 C_4_N_3_ discussed above, we can infer that the 2*s* and 2*p* orbitals of the nitrogen are approximately *sp*^3^-hybridized, while the 2*s* and 2*p* orbitals of the carbon undergo *sp*^2^ hybridization. Two N *sp*^3^-hybridized orbitals overlap significantly with the *sp*^2^-hybridized orbitals of two adjacent C_N_ atoms, forming strong *σ* bonds and corresponding σ* anti-bonding states. Another *sp*^3^-hybridized orbital of nitrogen is occupied by a lone pair of electrons, leaving one hybridized orbital singly occupied by one electron. The lone pair electron cloud, being unbound by bonding orbitals, exhibits higher energy and expanded volume. Consequently, the energy level of nitrogen unpaired electron is elevated due to repulsion from the lone pair of electrons. In the local C_C_-3C_N_ structure, the three *sp*^2^ orbitals of C_C_ overlap, respectively, with one *sp*^2^ orbital from the three C_N_ atoms, forming three *σ* bonds and three σ* anti-bonding states, leaving a C_C_ 2*p*_z_ orbital occupied by a single electron. For the C_N_ atom, after it forms three *σ* bonds and σ* anti-bonding states with a neighboring carbon and two nitrogen atoms through their respective overlapping hybrid orbitals, the C_N_ also retains one unpaired electron. Due to the higher electronegativity of nitrogen (Pauling scale 3.04) compared to carbon (2.55), the bonding electron cloud in the C_N_-N bond shifts towards the N. Consequently, besides the partial C_N_-N *σ* bonding electronic states shifting to a lower energy region (corresponding to the *σ*_1_ energy region in Figure 2a,c), the singly occupied 2*p*_z_ orbital level of C_N_ is lowered relative to that of C_C_. Therefore, the singly occupied orbital energy levels of C_N_ and N are closer to each other than those of C_N_ and C_C_. Most of the singly occupied states of C_N_ overlap with those of the two N atoms, while the remaining overlap with those of C_C_, forming *π* bonds and π* anti-bonding states; thus, all singly occupied orbitals of C_N_ bond with surrounding atoms. Although the remaining singly occupied states of C_C_ and N are close in energy, they hardly overlap due to their distance and poor orbital matching, resulting in singly occupied states located near the spin-up VBM, which are the source of magnetic performance. The *σ* bonding and σ* anti-bonding states are significantly split, corresponding to the lower energy region of the VB and the higher energy region of the CB, respectively (see Figure 2a–c). In contrast, the *π* and π* orbital energy levels are less split, corresponding to the higher energy region of the VB and the lower energy region of the CB (see Figure 2a–c). To clearly distinguish the bonding and anti-bonding states between the C_N_-N bond and the C_C_-C_N_ bond, we divided the 2*s* and 2*p* PDOSs of the C_C_, C_N_, and N in Pca21 C_4_N_3_ into different energy regions. As shown in Figure 2a–c, from low- to high-energy regions in the VB, there are *σ* bonding, *π* bonding, and non-bonding regions, while in the spin-up CB, from low- to high-energy regions, there are π* anti-bonding and σ* anti-bonding regions. The non-bonding region of the VB is mainly composed of the lone pair electrons on N and the single electron states on C_C_ and N, which determine the VBM. The *π* bonding energy region is divided into two segments, i.e., the *π*_1_ low-energy region and the *π*_2_ high-energy region. The *π* bonding electrons of the C_C_-C_N_ primarily contribute to the *π*_2_ energy region, while those of the C_N_-N are distributed in both the *π*_1_ and *π*_2_ energy regions, with N 2*p* electrons contributing more to *π*_1_ energy regions. In the *σ* bonding energy region, the *σ* bonding electrons of the C_C_-C_N_ mainly reside in the *σ*_2_ energy region, whereas those of the C_N_-N are spread across both the *σ*_1_ and *σ*_2_ energy regions. The CB is divided into the $\pi_{1}^{*}$, $\pi_{2}^{*}$, and σ* energy regions. The $\pi_{1}^{*}$ energy region mainly comprises the 2*p* states of N and C_N_, while $\pi_{2}^{*}$ primarily consists of the 2*p* states of C_C_ and C_N_ with a minor contribution from N 2*p*. The σ* energy region is located in the high-energy part of the CB. The spin-up CBM is determined by the π* state of C_N_-N, whereas the spin-down CBM is governed by the non-bonding 2*p* states of C_C_ and N. From Figure 2a,b, it is evident that the contribution of the 2*p* electrons of a single C_C_ to the magnetic moment is greater than that of a single N. Furthermore, the presence of N 2*s* states in the VB non-bonding, *π* bond, and *σ* bond regions and the appearance of 2*s* states of C_C_ and C_N_ in the VB low-energy (*σ*) region confirm the hypothesis of orbital hybridization for C and N. According to the role in orbital hybridization and bonding, we find the 2*s* orbital does not contribute to the magnetic moment; therefore, we will only analyze the 2*p* states of each system in subsequent discussions to simplify the analysis.

Bader charge analysis quantitatively illustrates the net electron transfer number between bonding atoms due to differences in electronegativity. In Pca21 C_4_N_3_, one C_N_ has a net loss of 1.06 *e*, while the nitrogen atom has a net gain of 1.06 *e*. This indicates that the C_N_-N bond has both ionic and covalent components. The net electron transfer for C_C_ is zero, indicating that the bonds formed between C_C_ and C_N_ are covalent bonds. The electron localization function (ELF) plot provides a more intuitive view of the bonding situation in the Pca21 C_4_N_3_ monolayer due to electron redistribution. Figure 2d clearly illustrates that the bonding electrons around C_C_ are concentrated along the C_C_-C_N_ bonds, showcasing *D*_3_ symmetry. Additionally, the bonding electrons are focused along the C_N_-N bond, with the lone pair electrons located at the apex of the ∠C_N_NC_N_ angle. Notably, the lone pair electrons of the three N in the six-membered microcycles are not coplanar, which implies that the singly occupied orbitals of the three nitrogen atoms are also asymmetrical. This may be due to the repulsion between the lone pair electrons on different nitrogen atoms, causing nitrogen atoms to stay as far apart as possible. Moreover, these lone pair electrons also influence the C_N_-N bond electron distribution (see Figure 2d), agreeing well with the highly corrugated structure and implying that the 2*p* orbitals provided by nitrogen atoms at different positions for bonding are not the same.

**Figure 3:**
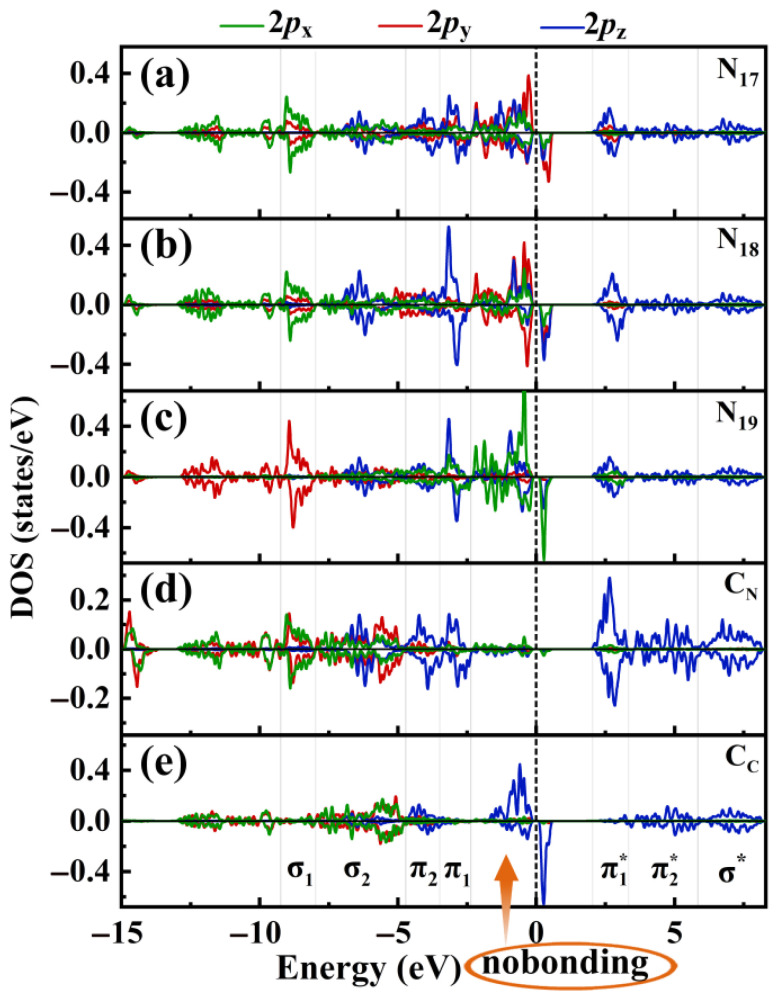
The spin-resolved PDOS of the 2*p*_x_, 2*p*_y_, and 2*p*_z_ for N (designated as (**a**) N_17_, (**b**) N_18_, and (**c**) N_19_; see Figure S1), and (**d**) C_N_, and (**e**) C_C_ in the Pca21 C_4_N_3_ monolayer. For C_C_, C_N_, and N, 2*p*_x_, 2*p*_y_ and 2*p*_z_ are represented by green, red, and blue lines, respectively.

Based on the analysis described above, we recognize that the lone pair electrons on nitrogen atoms possess high energy and significant spatial extension. This characteristic not only impacts the energy levels of the singly occupied orbitals of N but also alters the composition of the singly occupied orbitals of N. To further investigate the origin and mechanism of magnetism, we analyzed the PDOSs of the 2*p*_x_, 2*p*_y_ and 2*p*_z_ orbitals for three N atoms (designated as N_17_, N_18_, and N_19_, see Figure S1) of the six-membered microcycles in the Pca21 C_4_N_3_ monolayer, as well as those of the C_C_ and C_N_ atoms. The composition of the singly occupied orbitals and lone pair orbitals of the three N atoms (N_17_, N_18_, and N_19_), varies significantly (see Figure 3a–c). The lone pair and unpaired electron orbitals of N_17_ are primarily composed of 2*p*_x_, 2*p*_y_, and 2*p*_z_ orbitals, with a larger proportion of the 2*p*_y_ orbital. For N_18_, these electron orbitals are also formed by 2*p*_x_, 2*p*_y_, and 2*p*_z_ orbitals, but with a higher proportion of the 2*p*_y_ and 2*p*_z_ orbitals. In contrast, the N_19_ lone pair and unpaired electron orbitals mainly derive from the 2*p*_x_ and the 2*p*_z_ orbitals. The singly occupied states of the N atom located near the VBM serve as a part of the source of magnetic moment. As shown in Figure 3d, the 2*p*_z_ orbitals of C_N_ engage entirely in forming *π* and π* anti-bonding states, with the π* state contributing to the spin-up CBM. Figure 3e displays that the 2*p*_z_ orbital of C_C_ partially forms bonds, with the non-bonding occupied C_C_ 2*p*_z_ state located near the VBM as an additional source of magnetism, and the non-bonding unoccupied C_C_ 2*p*_z_ corresponds to the spin-down CBM. Additionally, the C_C_ *σ* bonding states are composed of 2*p*_x_ and 2*p*_y_ orbitals, indicating that these orbitals participate in *sp*^2^ hybridization.

### 2.2. The Electronic and Magnetic Modulation of Pca21 C_4_N_3_ Monolayer by Gas Adsorption

#### 2.2.1. Stability and Magnetic Property of Pca21 C_4_N_3_ Monolayer with Nitric Oxide (NO) or Oxygen (O_2_) Adsorption

To broaden the Pca21 C_4_N_3_ monolayer’s application range, gas adsorption is used to modulate its magnetic properties. Gas adsorption can be classified into physisorption and chemisorption. Physisorption is achieved through van der Waals forces, while chemisorption relies on the formation of chemical bonds. Based on the structural characteristics of the Pca21 C_4_N_3_ monolayer (see Figure S1), five possible adsorption sites were considered: **N** (positioned above the N atom), **C_N_** (positioned above the C_N_ atom), **C_C_** (positioned above the C_C_ atom), **R1** (located above the center of the six-membered microcycle), and **R2** (located above the center of the twelve-membered microcycle). The gas molecules (NO or O_2_) are initially oriented perpendicular to the monolayer surface. According to Equation 1, the adsorption energy (*E*_ads_) for each adsorption configuration was calculated.

**Figure 4:**
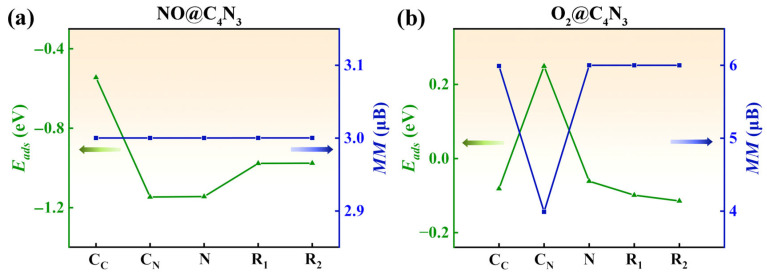
The adsorption energy and net magnetic moment of (**a**) NO@C_4_N_3_ systems and (**b**) O_2_@C_4_N_3_ systems at different adsorption sites. The green lines denote adsorption energy, and the blue lines indicate the values of the magnetic moment.

**Figure 5:**
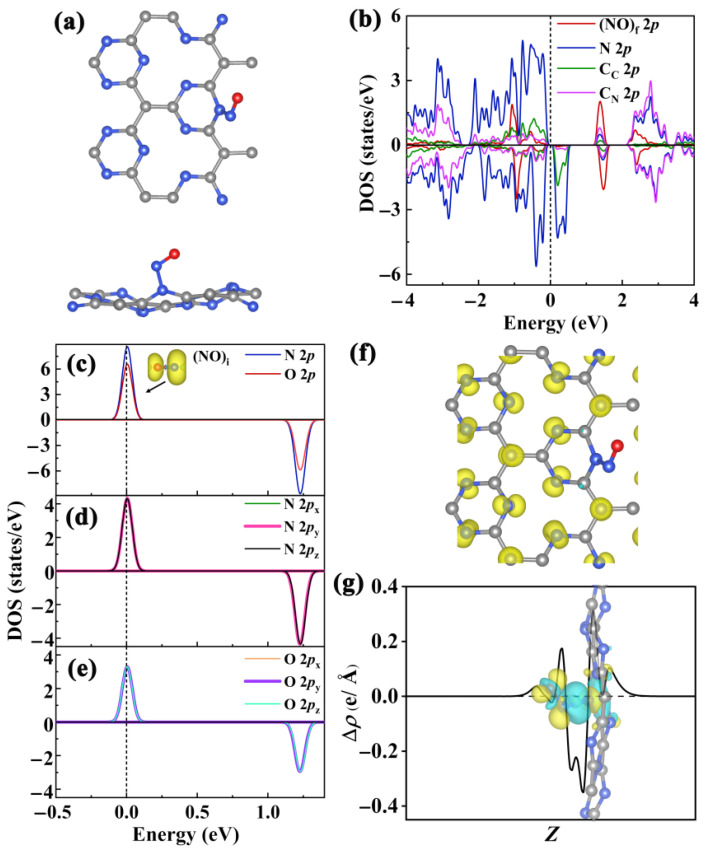
(**a**) The top (upper) and profile (lower) perspectives of the optimized configuration of the NO@C_4_N_3_ system. (**b**) The spin-resolved PDOS of 2*p* for (NO)_f_, C_C_, C_N_, and N in the NO@C_4_N_3_ system. The 2*p* of (NO)_f_ is denoted by red lines, and the C_C_ 2*p*, C_N_ 2*p*, and N 2*p* are denoted by blue, green, and pink lines, respectively. (**c**) In (NO)_i_, the spin-resolved PDOS of N 2*p* and O 2*p* is represented by blue and red lines, respectively. The inset displays the spatial distribution of spin-up π* orbitals for (NO)_i_. In (NO)_i_, (**d**) the PDOS of N 2*p*_x_, 2*p*_y_, and 2*p*_z_ is represented by green, rose, and black lines, respectively, and (**e**) the PDOS of O 2*p*_x_, 2*p*_y_, and 2*p*_z_ is represented by orange, purple, and cyan lines, respectively. (**f**) The views of the 3D isosurfaces (iso-value of 0.01 e/Å^3^) of net magnetization density for the NO@C_4_N_3_. (**g**) Integrals of CDD along the *z* direction for the NO@C_4_N_3_ system. The inset depicts the CDD distributions, with yellow regions representing electron accumulation and cyan regions indicating electron depletion. The isosurface value is established at 5.00 × 10^−3^ e/Å^3^. Red spheres denote O atoms, which also applies to the subsequent Figure 7.

For the NO adsorption system (NO@C_4_N_3_), the comparison of the *E*_ads_ values shown in Figure 4a reveals the most stable adsorption configuration (**C_N_** adsorption site), with the smallest *E*_ads_ value of −1.14 eV. The configuration of the NO@C_4_N_3_ system after being fully optimized is shown in Figure 5a. For convenience, NO is labeled as (NO)_i_ before adsorption and as (NO)_f_ after adsorption. Similarly, this notation applies to O_2_, with (O_2_)_i_ before adsorption and (O_2_)_f_ after adsorption. In Figure 5a, the nitrogen atom of (NO)_f_ (N_ad_) is bonded with the nitrogen atom from the substrate (N_sub_), and the value of ∠N_sub_N_ad_O is 114.7°. The length of the N_sub_-N_ad_ bond is 1.45 Å, and the N-O bond length increases from 1.17 Å before adsorption to 1.21 Å after adsorption. The net magnetic moment of the NO@C_4_N_3_ system is 2.99 μ_B_ at each adsorption site, which is less than the magnetic moment of pristine Pca21 C_4_N_3_ (4.00 μ_B_). According to the Van Hove equation (Equation (2)), the desorption temperature for NO is determined to be 521.8 K, which is much higher than the T_C_ (247 K) of the Pca21 C_4_N_3_ monolayer [32]. This implies that within T_C_, the adsorption of NO may lead to a reduction in the magnetic signal of Pca21 C_4_N_3_. Thus, the phenomenon of the weakening of magnetism within Tc could be judged as a necessary condition for NO adsorption on Pca21 C_4_N_3_. Therefore, Pca21 C_4_N_3_ has the potential to be developed as a NO gas sensor.

For the O_2_ adsorption system (O_2_@C_4_N_3_), according to the adsorption energy values, the stability order of the five adsorption sites is **C_N_** < **N** < **C_C_** < **R1** < **R2**. As shown in Figure 4b, the *E*_ads_ of the **C_N_** adsorption site is positive, indicating that this configuration does not exist. The *E*_ads_ values for the remaining four adsorption sites are negative, ranging from −0.06 to −0.11 eV. Among them, the **R2** adsorption site is the most stable, with an *E*_ads_ value of −0.11 eV. After optimization, the (O_2_)_f_ molecule is almost laid flat above the substrate. Based on the adsorption energy and the distance (2.79 Å) between the (O_2_)_f_ molecules and the Pca21 C_4_N_3_, it can be determined that they are physically adsorbed. Interestingly, the net magnetic moment of the O_2_@C_4_N_3_ system at each adsorption site increases to 6.00 μ_B_, increasing to 50% of the original sample.

Some previous studies [37,38,39] have highlighted the potential for stacking-induced functionality in similar crystalline materials, suggesting that interlayer interactions could provide further tunability. In order to investigate how stacking Pca21 C_4_N_3_ layers affects adsorption dynamics and magnetic responses in a system of NO or O_2_ adsorbed on C_4_N_3_ bilayers, we considered two patterns for the NO or O_2_ molecule adsorption, namely, interface and surface adsorption. For the interface adsorption, the gas molecule should cross the C_4_N_3_ monolayer to arrive at the interface region. As plotted in Figure S2a,b, it separately requires up to 6.04 eV and 3.72 eV for NO and O_2_ gas molecules to penetrate the interlayer through the macrocycle of the Pca21 C_4_N_3_ monolayer, indicating this mode of interface adsorption can be ruled out. As for the case of surface adsorption, based on our previous work [32], we established bilayer systems with three stacking configurations (AA, AB, and AC) to adsorb NO and O_2_ gas molecules, respectively (see Figure S3), and calculated the corresponding adsorption energies [37,38,39] and magnetic moments, which are listed in Table S1. On the Pca21 C_4_N_3_ bilayer with AA, AB, and AC stacking configurations, the adsorption energies of the NO gas molecule are −1.26, −0.85, and −1.07 eV, respectively, while those of the O_2_ gas molecule are 0.66, −0.11, and −0.44 eV, respectively. This indicates that interlayer interactions could provide tunability for the surface gas adsorption strength. However, interlayer interactions have a very weak effect on the net magnetic moments of these adsorption systems. The net magnetic moments of the systems with a NO gas molecule adsorbed on the C_4_N_3_ bilayer with the three stacking configurations are all almost 7.00 μ_B_; meanwhile, the ones with O_2_ gas molecule adsorption systems are all 10.00 μ_B_. The bilayer systems with adsorbed NO and O_2_ gas molecules have retained the ferromagnetic coupling characteristics of the pure bilayer systems [32]. Moreover, for the most stable adsorption configurations on the Pca21 C_4_N_3_ bilayer (NO on AA stacking pattern and O_2_ on AC stacking pattern), the adsorption energies are similar to those of the Pca21 C_4_N_3_ monolayer. The change in magnetic moment for these most stable adsorption configurations on the Pca21 C_4_N_3_ bilayer is also similar to that of the monolayer system when compared to the pure bilayer. For example, the O_2_ adsorption makes the magnetic moments of both the bilayer and monolayer systems increase by 2.00 μ_B_. Therefore, in the following study on the magnetic regulation mechanism, we do not discuss the bilayer situation separately.

#### 2.2.2. Electronic Structure of NO@C_4_N_3_ System

As shown in Figure 5b, the spin-down *E*_g_ of the most stable NO@C_4_N_3_ slightly decreases from 0.36 eV to 0.22 eV, with the spin-down CBM still originating from the 2*p* states of C_C_ and N. Meanwhile, the spin-up *E*_g_ significantly decreases from 2.24 eV to 1.36 eV. This notable change in the spin-up bandgap is due to the CBM of the NO@C_4_N_3_ system being composed of the 2*p* states of (NO)_f_ as well as the N 2*p* and C 2*p* states. The magnetic property of the NO@C_4_N_3_ system still primarily originates from the N 2*p* and C_C_ 2*p* state electrons. In order to study the magnetic moment distribution and the mechanism of magnetic changes in the NO@C_4_N_3_ system, the PDOS, spatial spin density distribution, charge difference density (CDD), and Bader charge of NO and the most stable NO@C_4_N_3_ system were systematically investigated. As shown in Figure 5c, the magnetic moment of (NO)_i_ is 1.00 μ_B_, primarily originating from the partially occupied 2*p* states of N and O below the Fermi level, with the contribution from the N 2*p* states being greater than that from the O 2*p* states. The N 2*p* states are composed of 2*p*_y_ and 2*p*_z_ (Figure 5d), while the O 2*p* states also consist of 2*p*_y_ and 2*p*_z_ (Figure 5e). Figure 5f illustrates the magnetic moment distribution of the NO@C_4_N_3_ system. The spatial spin density for (NO)_f_ and N_sub_ vanishes, contributing zero to the magnetic moment. The spatial spin density around the C_C_ atoms near N_sub_ (C_C-near_) decreases, indicating a reduced contribution to the magnetic moment from these C_C-near_ atoms. Meanwhile, the spin density distribution in the regions far from N_sub_ remains virtually unaffected, continuing to be dispersed on the C_C_ and N atoms. The CDD plot illustrates the changes in charge distribution induced by the formation of the new N_sub_-N_ad_ chemical bond. As depicted in Figure 5g, certain regions experience an increase in electron density (yellow areas), while other regions show a decrease (cyan areas). Notably, the electron density decreases in the bonding region of N_sub_-N_ad_, whereas the non-bonding regions of N_sub_, N_ad_, and O exhibit an increase in electron density. The decrease in electron density in the N_sub_-N_ad_ bonding region may be attributed to the fact that, before bonding with N_ad_, this region was primarily occupied by the lone pair electrons, *π* electrons, and single electrons of N_sub_. After bonding, the orbitals of the lone pair electrons change. Although this region is now a bonding area occupied by two bonding electrons, the number of electrons in this region is reduced compared to its original state, thus appearing as an electron-depleted area. The increase in electron density in some non-bonding regions of N_sub_, N_ad_, and O may be attributed to these areas becoming occupied by lone pair electrons. Near the N_sub_ of the substrate, electron accumulation (yellow areas) and depletion (cyan areas) indicate that NO adsorption causes electron transfer, redistributing electron density and forming stable chemisorption. Although the N_sub_-N_ad_ bond is formed by the same element, Bader charge analysis still shows a transfer of 0.058 e from the substrate to (NO)_f_. This can be attributed to the differences in electronegativity, namely, O (Pauling scale: 3.44) > N > C. The bonding electron cloud of the C-N_sub_ bond shifts towards N_sub_, while that of the N_ad_-O bond shifts towards O. As a result, the electronegativity of N_ad_ is slightly higher than that of N_sub_. When forming the N_sub_-N_ad_ bond, the overall effect is the net electron transfer from N_sub_ to N_ad_. The reason for the reduction in the magnetic moment of the NO@C_4_N_3_ system seems to differ from that of TCNQ-doped CrI_3_. For TCNQ-CrI_3_, the doping holes fill the half-occupied *t*_2g_ orbitals [40]. However, the mechanisms are similar: adsorption or doping leads to single-electron pairing, resulting in a reduced magnetic moment.

**Figure 6:**
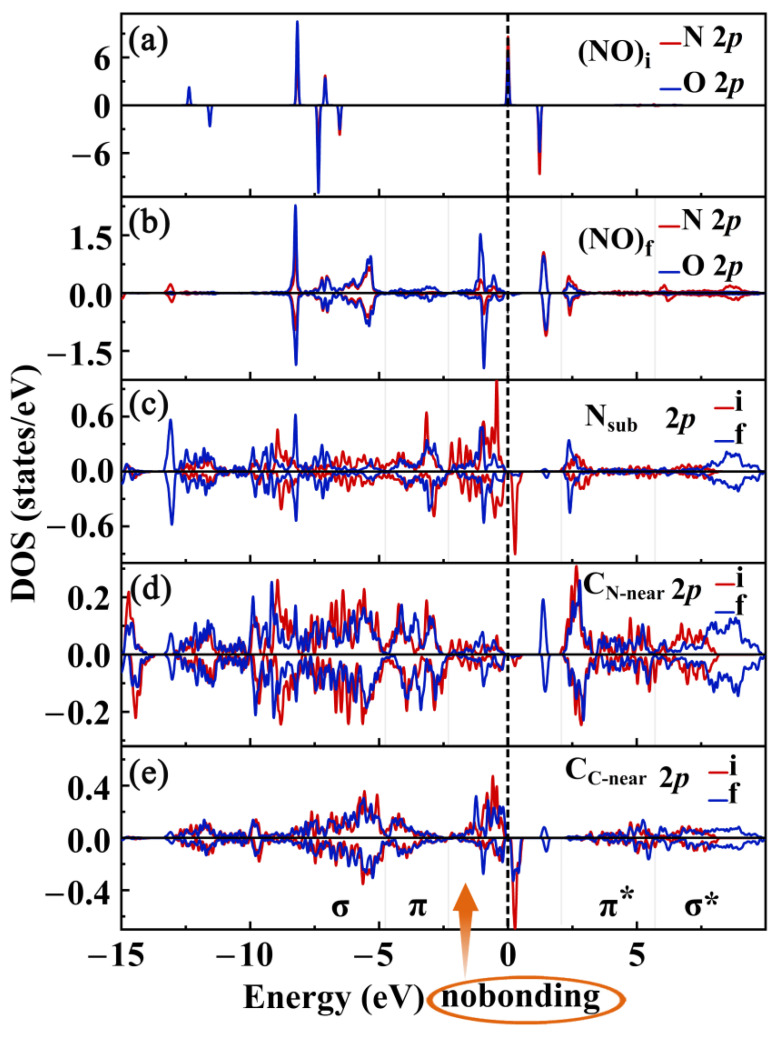
The spin-resolved PDOS of N 2*p* (denoted with red lines) and O 2*p* (denoted with blue lines) in (**a**) (NO)_i_ and (**b**) (NO)_f_; (NO)_i_ and (NO)_f_ denote NO before and after adsorption on Pca21 C_4_N_3_, respectively. The spin-resolved PDOS of 2*p* for N_sub_ (**c**), C_C-near_ (**d**), and C_N-near_ (**e**) in systems; the red lines labeled with **i** denote the 2*p* sates before NO adsorption, and the blue lines labeled with **f** represent the 2*p* after NO adsorption. N_sub_ refers to the N in the C_4_N_3_ substrate bonded with the NO molecule, C_C-near_ is C_C_ adjacent to N_sub_, and C_N-near_ is C_N_ neighboring N_sub_.

To further investigate the mechanism behind the magnetic changes in the NO@C_4_N_3_ system, we calculated the PDOS of 2*p* orbitals for NO before and after adsorption, as well as the PDOS of 2*p* orbitals for N_sub_, C_C-near_, and C_N-near_ (the C_N_ adjacent to N_sub_) within the NO@C_4_N_3_ system (see Figure 6). In (NO)_i_, the 2*p*_x_ orbitals of N and O have a significant head-to-head overlap along the bond axis, forming a strong *σ* bond occupied by electrons. Meanwhile, the 2*p*_y_ and 2*p*_z_ orbitals of N and O overlap side-by-side, forming two *π* bonding orbitals occupied by electrons and two partially occupied π* anti-bonding orbitals. The *π* bonding orbitals have a greater contribution from the O 2*p* states, while the π* anti-bonding orbitals have a greater contribution from the N 2*p* states (see Figure 5c and Figure 6a). The energy levels of the two spin-up π* orbitals are degenerate and lower than those of the two spin-down degenerate π* orbitals. The two spin-up π* orbitals are occupied by only one electron, resulting in a magnetic moment of 1.00 μ_B_. The bonding and anti-bonding states of the (NO)_i_ molecule exhibit strong localization, and the Fermi level divides the spin-up π* states (see Figure 5c and Figure 6a). After adsorption, due to the formation of the N_sub_-N_ad_ bond, the orbitals of N_ad_ can be approximated as sp^3^-hybridized. The interaction between N_ad_ and O can be understood as consisting of one *σ* bond and one *π* bond, along with an orbital containing a lone pair of electrons and another orbital occupied by a single electron. The overlap of these two singly occupied orbitals forms a *π* bond occupied by two electrons and an empty π* state. Therefore, after adsorption, the magnetic properties of (NO)_f_ disappear. In (NO)_f_, the lone pair electrons on N_ad_ and O are located at the ∠N_sub_N_ad_O angle and on O (as shown in Figure 5g). Compared to the PDOS of (NO)_i_ (Figure 6a), due to changes in bonding properties and the influence of the substrate, the 2*p* states of N_ad_ and O become more delocalized (see Figure 6b). Figure 6c shows the distribution of the N_sub_2*p* states and provides information about its bonding. After adsorption, in addition to containing a lone pair of electrons, N_sub_ forms three *σ* bonds with surrounding atoms, the N_sub_-N_ad_ bond and two N_sub_-C_N-near_ bonds, thereby achieving an eight-electron stable state. Consequently, N_sub_ contributes zero magnetic moment to the NO@C_4_N_3_ system. Constrained by these three strong *σ* bond orbitals, the corresponding *σ* bonding electrons shift to lower energy levels. Due to the absence of its own non-bonding single electron, the lone pair electrons on N_sub_ experience a reduction in energy. The position and shape of this lone pair orbital are altered due to the influence of the formed N_sub_-N_ad_ bond (see Figure 5g). After adsorption, the atoms providing paired electrons for the singly occupied orbital of C_N-near_ decrease from three (two N and one C_C_) to two (C_C-near_ and N). Consequently, the proportion of paired electrons from these two atoms increases, reducing the contribution of C_C-near_ to the system’s magnetism (see Figure 6e). Under the influence of NO chemisorption, C_N-near_ 2*p* states (see Figure 6d), N_sub_ 2*p* states (see Figure 6c), and C_C_ 2*p* states (see Figure 6e) are induced below the original system’s CBM. These induced 2*p* states, along with the 2*p* states of (NO)_f_, form the spin-up CBM of the NO@C_4_N_3_ system, significantly reducing the spin-up bandgap. In summary, the N_sub_-N_ad_ chemical bond is formed between the (NO)_f_ and the substrate, leading to significant changes in the bonding nature and electron distribution of N_sub_, N_ad_, and O. As a result, (NO)_f_ and N_sub_ no longer have orbitals occupied by unpaired electrons and thus do not contribute to the magnetic moment. Additionally, the contributions of C_C-near_ to the magnetic moment are also reduced. Furthermore, the CBM of the adsorption system is mainly composed of the 2*p* state of (NO)_f_ and its induced electronic states, resulting in a significant reduction in the spin-up bandgap. This reduced bandgap helps to enhance the photoresponse to some extent.

#### 2.2.3. Electronic Structure of O_2_@C_4_N_3_ System Through Magnetic Coupling Between O_2_ and C_4_N_3_ Pca21 C_4_N_3_ Monolayer

**Figure 7:**
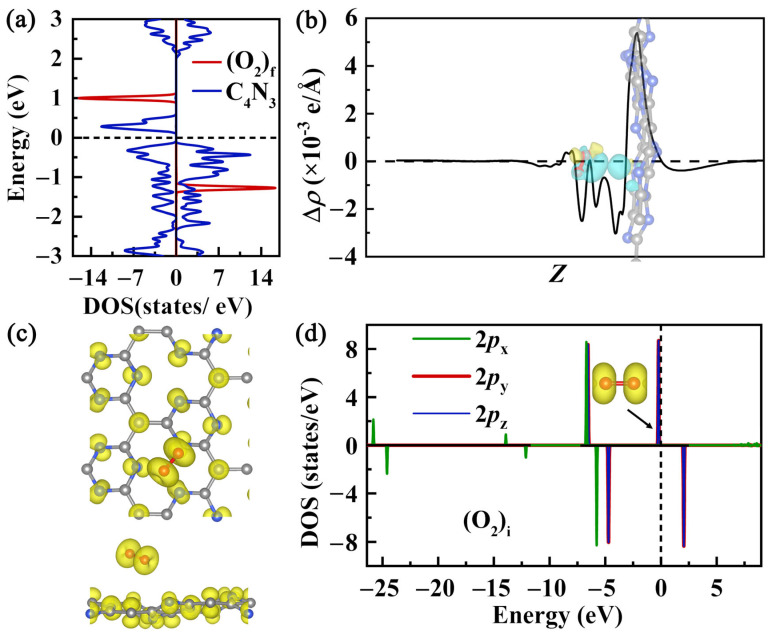
(**a**) The PDOSs for (O_2_)_f_ and C_4_N_3_ in the O_2_@C_4_N_3_ system, which are represented by blue and red lines, respectively. (**b**) Integrals of CDD along the *z* direction for the O_2_@C_4_N_3_ system. The inset depicts the CDD distributions, with yellow regions representing electron accumulation and cyan regions indicating electron depletion. The isosurface value is established at 2.00 × 10^−3^ e/Å^3^. (**c**) The top (upper) and profile (lower) views of the 3D isosurfaces (the iso-value is 1.15 × 10^−2^ e/Å^3^) of net magnetization density for the O_2_@C_4_N_3_ monolayer. (**d**) The spin-resolved PDOSs of the 2*p*_x_, 2*p*_y_, and 2*p*_z_ for (O_2_)_i_, which are represented by green, red, and blue lines, respectively. The inset displays the spatial distribution of spin-up π* orbitals for (O_2_)_i_.

To investigate the impact of O_2_ adsorption on the electronic structure and magnetic distribution of the Pca21 C_4_N_3_ monolayer, we studied the DOS, CDD, and spin density distribution of the most stable O_2_@C_4_N_3_ system. Compared to the pristine Pca21 C_4_N_3_, the spin-up *E*_g_ of the O_2_-adsorbed system slightly decreases from 2.24 eV to 2.22 eV, while the spin-down *E*_g_ slightly increases from 0.36 eV to 0.42 eV. As shown in Figure 7a, the contribution of the O orbitals in both spin channels is primarily concentrated in the deep-level regions (below −1.00 eV and above 1.00 eV). Near the Fermi level, the DOS peak for (O_2_)_f_ is sharp and narrow, exhibiting strong localization, which indicates that it is minimally affected by the substrate. This also indirectly suggests that the interaction between the O_2_ gas and the substrate is mainly due to physical adsorption. The CDD map (see Figure 7b) shows that weak interactions between O_2_ molecules and the substrate cause slight changes in the spatial electron distribution. There are small regions of electron accumulation (yellow areas) and depletion (cyan areas) on both the substrate and (O_2_)_f_. Compared with the magnetic moment distribution of the pristine Pca21 C_4_N_3_ monolayer (see Figure 1b) and (O_2_)_i_ (see the inset of Figure 7d), the magnetic moment distribution of the substrate and (O_2_)_f_ (Figure 7c) is almost unaffected, with the magnetic moments still located on the (O_2_)_f_ molecules and the substrate’s magnetic moments still distributed on N and C_C_.

In order to investigate the interaction between O_2_ and the substrate, we calculated the PDOS of the 2*p*_x_, 2*p*_y_, and 2*p*_z_ orbitals of (O_2_)_I_; the spin density distribution of O_2_; and the PDOS of the 2*p* orbitals of the three N, three C, and six C_N_ atoms in the twelve-membered microcycle below the **R2** adsorption site. According to the PDOS of (O_2_)_i_ (Figure 7d), it can be inferred that the 2*p*_x_ orbitals of the two O atoms overlap head-to-head, resulting in a large energy splitting into *σ* bonding and σ* anti-bonding orbitals. The 2*p*_y_ and 2*p*_z_ orbitals of the two O atoms overlap side-by-side, forming two *π* bonding orbitals and two π* anti-bonding orbitals with smaller energy splitting. The energy levels of the two spin-up π* orbitals are degenerate and lower than those of the two spin-down degenerate π* orbitals. The two spin-up π* orbitals are occupied by two electrons, leaving the two spin-down π* anti-bonding orbitals empty, resulting in a magnetic moment of 2.00 μ_B_. The 2.00 μ_B_ magnetic moment of (O_2_)_i_ is uniformly distributed in the spin-up π* anti-bonding orbitals (see the inset in Figure 7d). The highest occupied molecular orbital (HOMO) energy level of (O_2_)_i_ is −6.78 eV. After adsorption, due to the magnetic attraction from the substrate, the HOMO energy level of (O_2_)_f_ shifts down by 0.04 eV. Compared to the initial state, the 2*p* PDOSs of (O_2_)_f_ for spin-up and spin-down π* anti-bonding orbitals show increased peaks near the Fermi level, but the broadening remains narrow (see Figure S4a), indicating strong localization of oxygen electronic states. At the substrate adsorption site, the PDOSs of 2*p* for the C and N atoms in the twelve-membered microcycle show almost no noticeable change (see Figure S4b–d). Theoretically, the 2*p* PDOS of these C and N atoms should exhibit a slight shift towards lower energy levels. However, due to the extremely weak magnetic interaction of the (O_2_)_f_ molecule, any changes in the PDOS are difficult to discern. This further demonstrates that the interaction between (O_2_)_f_ and the substrate is physisorption.

From the above analysis, it is evident that in the pristine Pca21 C_4_N_3_ system, the combined contribution of three N atoms to the system’s magnetic moment is greater than that of one C_C_ (Figure 1a). However, the contribution of an individual C_C_ exceeds that of a single N atom (Figure 2a,b). Consequently, the qualitative order of the net magnetic moment for the five adsorption sites on the substrate, from smallest to largest, can be determined as **C_N_** < **N** < **C_C_** < **R1** < **R2**. This order perfectly aligns with the stability sequence of the five adsorption sites. This implies that the interaction between the (O_2_)_f_ molecule and the substrate is primarily driven by magnetic interactions, specifically characterized by the repulsion between like magnetic poles and the attraction between opposite magnetic poles. For the most stable O_2_@Pca21 C_4_N_3_ system, the adsorption of O_2_ significantly enhances the system’s magnetic moment, increasing from 4.00 μ_B_ to 6.00 μ_B_, indicating that a ferromagnetic coupling may exist between the (O_2_)_f_ and the substrate. In order to confirm this point, we calculate the energy difference between the ferromagnetic state and antiferromagnetic states. The results show that the total energy of the system in the ferromagnetic state (*E*_FM_) is 0.08 eV lower than that in the antiferromagnetic state (*E*_AFM_), i.e., *E*_FM_ − *E*_AFM_ = −0.08 eV. The ferromagnetic coupling may also explain why the **R2** site is the most stable adsorption site for the O_2_ gas molecule. Specifically, when (O_2_)_i_ is positioned at the **R2** site, which has the largest magnetic moment among the five adsorption sites, the magnetic interaction is the strongest, resulting in the lowest adsorption energy and the most stable system.

## 3. Computation Details

The fundamental calculations were performed using density functional theory (DFT) with the Vienna ab initio Simulation Package (VASP) [41]. The exchange-correlation function was handled using the Perdew–Burke–Ernzerhof (PBE) form within the generalized gradient approximation (GGA) [42]. The projector augmented wave (PAW) method was used to model electron–ion interactions [43]. Van der Waals (vdW) corrections were included using the DFT-D3 method as described by Grimme [44,45]. A vacuum layer greater than 20 Å in the non-periodic direction prevented interactions between periodic images. Geometry optimizations involved relaxing every atom in the supercell, applying a force convergence criterion of 0.05 eV/Å and a total energy convergence of 10^−5^ eV. A 5 × 5 × 1 Monkhorst-Pack k-point mesh was employed to sample the Brillouin zone. The plane-wave basis set cutoff for the electron wave functions was fixed at 500 eV.

The adsorption energy $E_{\mathrm{ads}}$ for a single gas molecule on the Pca21 C_4_N_3_ monolayer is defined as

(1) $$E_{\mathrm{ads}}=E_{\mathrm{total}}-E_{\mathrm{gas}}-E_{C_{4}N_{3}}$$

where $E_{\mathrm{total}}$, $E_{\mathrm{gas}}$, and $E_{C_{4}N_{3}}$ are the total energy of the adsorption system, isolated NO gas molecule, and pristine Pca21 C_4_N_3_ monolayer, respectively. Based on the definition of adsorption energy, the lower the adsorption energy of the adsorption system, the higher its stability.

In order to evaluate the thermal stability and reversibility of the NO@C_4_N_3_ configuration in practical applications, the Van Hove equation was employed to estimate the desorption temperature *T*_d_, as indicated below:

(2) $$T_{d}=\left(\frac{\left|E_{ads}\right|}{k_{B}}\right){\left(\frac{\Delta S}{R}-\ln\frac{P}{P_{0}}\right)}^{-1}$$

where *R* represents the universal gas constant, $k_{B}$ stands for the Boltzmann constant, *E*_ads_ indicates the adsorption energy, *P* is the atmospheric pressure, *P* = *P*_0_ = 1 atm, and ∆*S* denotes the entropy change associated with the phase transition of NO from liquid to gas and its value of 210.8 J∙K^−1^∙mol^−1^ is obtained from the handbook in [46].

## 4. Conclusions

First-principles calculations were conducted to systematically investigate the magnetic mechanism and magnetic regulation of a 2D organic semiconductor Pca21 C_4_N_3_ monolayer by adsorbing magnetic polar NO or non-polar O_2_ gas molecules. The calculated result reveals that the magnetic moment of the Pca21 C_4_N_3_ monolayer with ferromagnetic stability originates from the C_C_ 2*p*_z_ and N 2*p* electrons due to the influence of the lone pair electrons on nitrogen atoms. For the most stable NO@Pca21 C_4_N_3_ system, the adsorption of NO pairs an unpaired electron from the substrate nitrogen atoms with a single magnetic electron in the NO molecule, forming a N_sub_-N_ad_ chemical bond. This interaction significantly reduces the system’s magnetic moment from 4.00 to 2.99 μ_B_, decreases the spin-down *E*_g_ from 0.36 to 0.22 eV, and the spin-up *E*_g_ greatly decreases from 2.24 to 1.36 eV. The desorption temperature for NO is 521.8 K, much higher than the T_C_ (247 K) of the Pca21 C_4_N_3_ monolayer. Therefore, NO gas adsorption can effectively modulate the electronic and magnetic properties of the Pca21 C_4_N_3_ monolayer, suggesting its potential application as a gas sensor by monitoring changes in magnetic and electronic properties. For the stable O_2_@Pca21 C_4_N_3_ system, no chemical bond is formed between (O_2_)_f_ and the substrate, and their weak interaction from magnetic pole attraction has a negligible effect on the electronic structure of the system. However, O_2_ exhibits ferromagnetic coupling with the substrate, enhancing the system’s magnetic moment from 4.00 to 6.00 μ_B_. Our comprehensive findings not only reveal the magnetic mechanism in the Pca21 C_4_N_3_ monolayer, but also uncover a simple and cost-effective method to regulate its magnetic moment, as well as its potential application as a NO gas sensor. These insights provide crucial theoretical foundations and research directions for emphasizing the significance of the Pca21 C_4_N_3_ monolayer in the realm of low-dimensional magnetic materials and expanding its application potential.

## Data Availability

Data are contained within the article and Supplementary Materials.

## Supplementary Materials

The following supporting information can be downloaded at https://www.mdpi.com/article/10.3390/molecules29215194/s1: Figure S1: Top and side views of the Pca21 C_4_N_3_ monolayer; Figure S2: The energy barriers for NO and O_2_ diffusion in the 12-membered macrocycles of Pca21 C_4_N_3_ monolayer; Figure S3: The top and side views of NO and O_2_ adsorbed on AA, AB, and AC stacking patterns for the Pca21 C_4_N_3_ bilayer; Figure S4: The spin-resolved PDOS of 2*p* for O_2_, and three N, three C_C_ and six C_N_ in the 12-membered microcycles at the R2 adsorption configuration; Table S1: The net magnetic moment and adsorption energy for NO and O_2_ adsorbed on C_4_N_3_ monolayer and bilayer with different stacking patterns.
