# Supplementary material for: Theoretical Study of the Magnetic Mechanism of a Pca21 C4N3 Monolayer and the Regulation of Its Magnetism by Gas Adsorption
Source: Molecules. 2024 Nov 2;29(21):5194. doi: 10.3390/molecules29215194 (PMC11547585; doi:10.3390/molecules29215194)
Supplement: Supplementary file 1 [file molecules-29-05194-s001.zip › molecules-3276930-supplementary.pdf]

Supporting Information

Theoretical Study of the Magnetic Mechanism of a Pca21 C_4N_3 Monolayer and the Regulation of Its Magnetism by Gas Adsorption

Dongqiu Zhao [1], Xiao Tang [2,*], Xueying Gao [1], Wanyan Xing [1], Shuli Liu [1], Huabing Yin [3] and Lin Ju [1,*]

[1] School of Physics and Electric Engineering, Anyang Normal University, Anyang 455000, China; dqzhao@aynu.edu.cn (D.Z.); 221101050@stu.aynu.edu.cn (X.G.); 221101039@stu.aynu.edu.cn (W.X.); 01958@aynu.edu.cn (S.L.)
[2] College of Science, Nanjing Forestry University, Nanjing 210037, China
[3] Joint Center for Theoretical Physics, Institute for Computational Materials Science, School of Physics and Electronics, Henan University, Kaifeng 475004, China; yhb@henu.edu.cn
* Correspondence: xiaotang@njfu.edu.cn (X.T.); julin@aynu.edu.cn (L.J.)

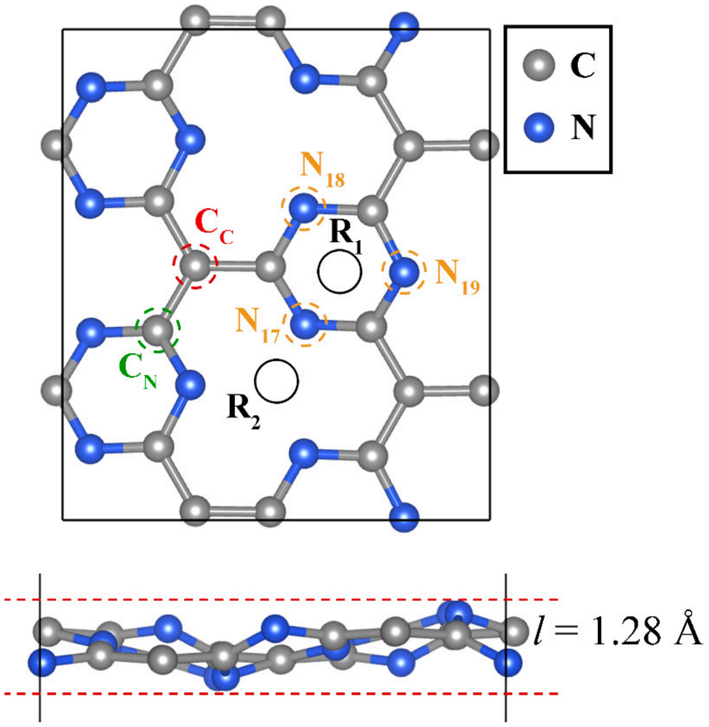

Figure S1. Top and side views of the Pca21 C_4N_3 monolayer. Blue spheres represent N atoms, and gray spheres indicate C atoms. The red dashed circle indicates C_C atoms, while the green dashed circle represents C_N atoms. The five adsorption sites: **N**, **C_N**, **C_C**, **R1**, and **R2** are the top sites above the N, C_N, C_C, R1, and R2, respectively. The orange dashed circles indicate three N atoms (designated as N_{17}, N_{18}, and N_{19}) in the 6-membered microcycles of the Pca21 C_4N_3.

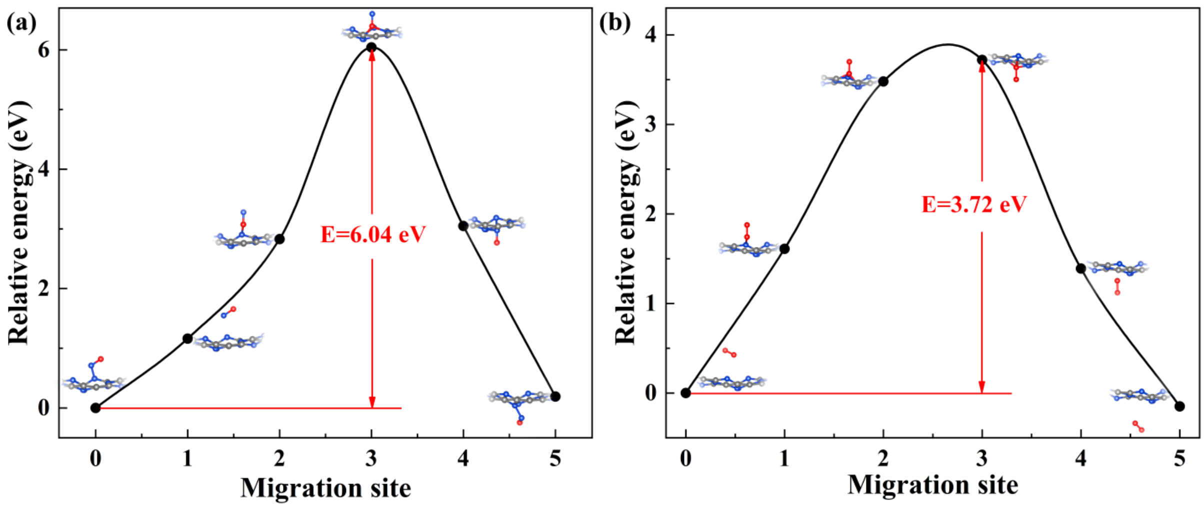

Figure S2. The energy barriers for (a) NO and (b) O_2 diffusion in the 12-membered macrocycles of a Pca21 C_4N_3 monolayer.

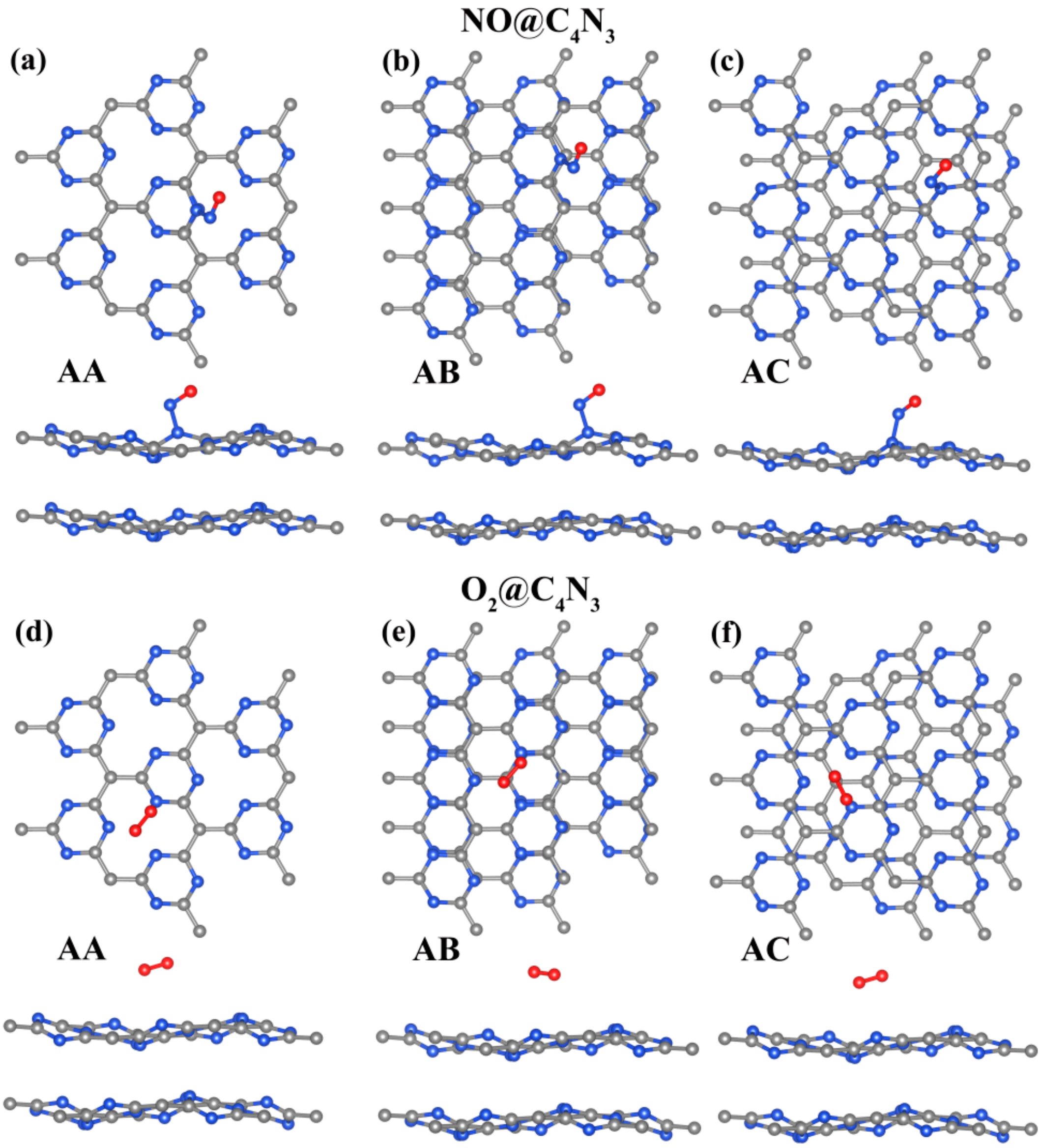

Figure S3. The top (upper) and side (lower) views of NO adsorbed on (a) AA, (b) AB, and (c) AC stacking patterns, and O_2 adsorbed on (d) AA, (e) AB, and (f) AC stacking patterns for the Pca21 C_4N_3 bilayer. AA stacking pattern: the C and N atoms of the upper layer recombined with the C and N atoms of the lower layer, respectively. AB stacking pattern: the C and N atoms of the upper layer are overlapped with the N and C atoms of the lower layer, i.e., the AB stacking pattern is shifted by one C-C bond distance with respect to the AA stacking pattern. AC stacking pattern: the C and N atoms of the upper layer are coincident with those of the lower layer, i.e., the AC stacking pattern is shifted by one C-N bond distance relative to the AB stacking pattern.

Table S1. The net magnetic moment (*MM*) and adsorption energy (E_{ads}) for NO and O_2 adsorbed on C_4N_3 monolayer and bilayer with different stacking patterns.

Pca21 C_4N_3	monolayer	AA	AB	AC
E_{ads} (eV) for NO adsorbed systems	-1.14	-1.26	-0.85	-1.07
E_{ads} (eV) for O_2 adsorbed systems	-0.11	0.66	-0.11	-0.44
MM (μ_B) for pure systems	4.00	8.00	8.00	8.00
MM (μ_B) for NO adsorbed systems	2.99	7.00	6.97	7.00
MM (μ_B) for O_2 adsorbed systems	6.00	10.00	10.00	10.00

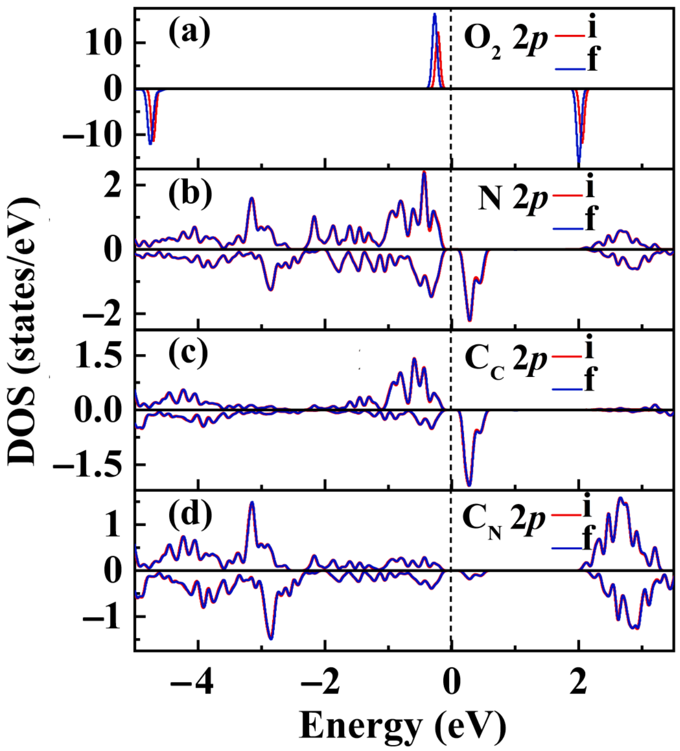

Figure S4. The spin-resolved PDOS of 2*p* for (a) O_2, and (b) three N, (c) three C_C and (d) six C_N in the 12-membered microcycles at the **R2** adsorption site of the Pca21 C_4N_3. The red lines labeled with **i** indicate the 2*p* states before O_2 adsorption, while the blue lines labeled with **f** represent the 2*p* states after O_2 adsorption.
